# Role of Natural Compounds in Oxidative Stress and Inflammation Linked to Cardiometabolic Disorders: From Biochemical Aspects to Clinical Evidences

**DOI:** 10.1155/2018/1479309

**Published:** 2018-05-08

**Authors:** Cristiana Caliceti, Paola Rizzo, Mariateresa Giuliano

**Affiliations:** ^1^University of Bologna, Bologna, Italy; ^2^University of Ferrara and GVM Care & Research, Maria Cecilia Hospital, Cotignola, Italy; ^3^University of Campania, Caserta, Italy

The most cost-effective preventive approach still remains diet and physical activity, also in people without a history of cardiovascular disease. However, lifestyle programs are often difficult to follow for long periods of time, and changes in dietary habits and physical activity sometimes are not enough to reduce risk parameters, such as hypercholesterolemia. In this context, an everyday approach utilizing dietary supplements, nutraceuticals, phytochemicals, and functional foods could improve blood lipid profile in humans and protect cells from oxidative stress and from damage related to inflammatory conditions. Since the prevention of cardiometabolic disorders is a fundamental strategy to decrease hospitalization and the health apparatus costs, a nutraceutical treatment could be, at least in part, a possible weapon to use. In this context, the scientific community has to adequately define the tolerability and safety of dietary supplements, either nutraceuticals or botanicals, as well as understand the precise mechanisms of actions and the risk/benefit ratio related to their assumption.

This special issue offers a selected and articulated overview of the examined topics. It contains seven papers, and the details were listed as follows:

J.-F. Feng et al. explored the therapeutic mechanism of *Dioscorea nipponica* (DN), a medicinal plant used to treat myocardial ischemia (MI), identifying the metabolites generated by intestinal microflora from DN and their cardioprotective efficacy. Results demonstrated that diosgenin, the main metabolite produced by rat intestinal microflora from DN, protects the myocardium against ischemic insult through increasing enzymatic and nonenzymatic antioxidant levels in vivo and by decreasing oxidative stress damage.

J. Tian et al. reviewed the use of herbal medicines for diabetes treatment, to prevent cardiovascular complications. Molecules and signal transduction pathways were critically analyzed, and specific effects of several compounds were highlighted.

Novel pharmacological targets have been investigated by Z. Wang et al. Original data indicated that TRPA1 might be an alternative pharmacological target to mitigate the detrimental cardiac effects induced by doxorubicin. Using an animal model, they demonstrated that blockage of TRPA1 can prevent cardiomyocyte apoptosis, reducing inflammation and oxidative and ER stress.

J. Tian et al. showed that *Ginkgo biloba* leaf extract (GBE) significantly attenuated cardiomyocyte apoptosis, collagen deposition, and inflammation in diabetic mice via inhibition of the p-JNK, CHOP, and caspase-12 pathways, as well as decreasing the serum levels of the proinflammatory cytokines (IL-6, IL-1*β*, and TNF-*α*). Blood glucose and lipid profiles were also regulated. These results suggested that GBE might be beneficial in the treatment of diabetic myocardial injury.

K.-H. Cho et al. studied policosanol with in vitro, in vivo (in female subjects), and ex vivo experiments to provide more substantial and concrete data on blood pressure-lowering effect. They showed that consumption of policosanol for 8 weeks in healthy female subjects lowered blood pressure and CETP activity via elevation of HDL/apoA-I contents and enhancement of HDL functionalities, including cholesterol efflux and insulin secretion, thus contributing to the prevention of aging-related diseases, hypertension, and stroke.

V. Valli et al. evaluated the changes in the expression of adipogenic markers (C/EBP*α*, PPAR*γ* variant 1 and variant 2, and GLUT4) in 3T3-L1 murine preadipocytes at four stages of the differentiation process and compared the effectiveness of sulforaphane, genistein, and docosahexaenoic acid in reducing lipid accumulation and modulating C/EBP*α*, PPAR*γ*1, PPAR*γ*2, and GLUT4 mRNA expression in mature adipocytes, showing that all bioactive compounds suppress adipocyte differentiation. Since obesity is characterized by excess body fat accumulation due to an increase in size and number of differentiated mature adipocytes, these results confirmed that several natural food constituents could be used as important agents in preventing or treating obesity.

N. Calabriso et al. exploited the role of hydroxytyrosol (HT), a well-known olive oil antioxidant on mitochondrial oxidative stress in phorbol myristate acetate- (PMA-) challenged endothelial cells. They observed that HT blunts endothelial dysfunction and pathological angiogenesis by ameliorating mitochondrial function, thus suggesting HT as a potential mitochondria-targeting antioxidant in the inflamed endothelium. The guest editors hope that the information provided in this special issue is useful and offers a scientific profile of the effects of some dietary supplements, nutraceuticals, and phytochemicals on cardiovascular diseases linked to oxidative stress and inflammation.

Finally, we would like to thank the authors for an excellent contribution of their research works, and we also very warmly acknowledged the reviewers for an excellent contribution of their valuable review results.

*Cristiana Caliceti* *Paola Rizzo* *Mariateresa Giuliano*

